# Scanning Electron Microscopy and X-Ray Microanalysis for Chemical and Morphological Characterisation of the Inorganic Component of Gunshot Residue: Selected Problems

**DOI:** 10.1155/2014/428038

**Published:** 2014-06-15

**Authors:** Zuzanna Brożek-Mucha

**Affiliations:** ^1^Faculty of Chemistry, Jagiellonian University, Ingardena Street 3, 30-060 Krakow, Poland; ^2^Department of Criminalistics, Institute of Forensic Research, Westerplatte Street 9, 31-033 Krakow, Poland

## Abstract

Chosen aspects of examinations of inorganic gunshot particles by means of scanning electron microscopy and energy dispersive X-ray spectrometry technique are presented. The research methodology of particles was worked out, which included a precise and repeatable procedure of the automatic detection and identification of particles as well as the representation of the obtained analytical data in the form of the frequencies of occurrence of particles of certain chemical or morphological class within the whole population of particles revealed in a specimen. On this basis, there were established relationships between the chemical and morphological properties of populations of particles and factors, such as the type of ammunition, the distance from the gun muzzle to the target, the type of a substrate the particles sediment on, and the time between shooting and collecting the specimens. Each of these aspects of examinations of particles revealed a great potential of being utilised in casework, while establishing various circumstances of shooting incidents leads to the reconstruction of the course of the studied incident.

## 1. Introduction

Inorganic components of gunshot residue (GSR), in particular, metallic particles originating from the ammunition primer, reveal characteristic properties and so provide themselves a high value evidence of using a firearm. Only few particles, of total mass no greater than 100 picograms, can be accepted as the evidence relating an individual with a shooting incident. The fundamental criterion of the identification of metallic gunshot particles is their chemical contents, that is, rarely occurring set of elements: lead, antimony, and barium as well as their morphology reflecting the kinetics of the processes undergoing during a gunshot, especially rapid cooling of droplets of the molten metals present in the expanding plum of products of the primer detonation and the propellant combustion.

As reported and reviewed by many, see, for example, Dalby et al. [1], formal classification scheme for gunshot particles being worked out and applied by the experts in Western Europe and USA since about 1980 on the basis of the experiences with traditional ammunition embraced a division into* unique* and* indicative* particles. Particles simultaneously containing lead, antimony, and barium (Pb-Sb-Ba) were classified into unique ones allowing stating, categorically, that they originated from a firearm discharge. Particles containing two- or one-component combinations of these elements (Pb-Sb, Pb-Ba, Sb-Ba, Pb, Sb, and Ba) as well as the ones containing barium, calcium, and silicon (Ba-Ca-Si) were classified into the class of indicative particles that enable one to infer on their origin from a firearm discharge with a high probability. However, since about 2003, experts resigned from the terms* unique* and* indicative* for the benefit of* characteristic* and* consistent with gunshot residue*, taking into account the publication of Torre et al. [2], who demonstrated the presence of a single particle containing lead, antimony, and barium and revealing an oval shape that resembled the morphology of GSR, however, originating from car brake pads. Thus, nowadays, one cannot identify a gunshot particle with certainty but with a high probability, similarly as any other material being a criminalistic microtrace. Nevertheless, the three-component particles, especially when numerously occurring in a sample, provide themselves a high value evidence of a gunshot. Establishing the presence of gunshot residue in the sample collected from hands or clothing of a person allows relating the person with a high or very high probability, either with shooting or being in the nearest vicinity of a firing gun or else getting in contact with an object highly contaminated with GSR.

Questions directed to a forensic chemist by the practitioners of administration of justice concerned also established the type of ammunition considering GSR particles as the only evidence being accessible for investigations. Despite the* cliché* that chemical analysis of GSR does not contribute to establishing the type of ammunition they originate from, the initial results of research on the chemical composition of GSR originating from ammunition by Mesko Metal Works, Skarżysko-Kamienna, Poland, revealed lack of particles containing, simultaneously, lead, antimony, and barium [3]. That demonstrated that chemical composition of the primer in this ammunition differed from chemical composition of primers in ammunition present on the market in other countries. This lead the author to the conclusion that the formal scheme of classification of GSR and so the system of the evaluation of the evidence might be inadequate for preparing reliable expert's reports and inspired to perform further research in this field of science.

The discrepancy between formal classification scheme of GSR and the possibility of detection of particles originating from ammunition primer types that are different from the traditional ones was also noticed by Romolo and Margot [4]. They proposed another individual attempt to the evaluation of the evidential value of metallic particles based on the mutual consistency of particles found in the items of certain case, called* case-to-case* approach, rather than comparing them with the arbitrary classification scheme. The majority of works published earlier concerned only the identification of lead, antimony, and barium particles and so the statement whether these particles are present in the studied material and whether they are distinguishable from particles of similar chemical contents but originating from different sources than a firearm discharge.

Further important questions concerned the possibility of establishing the mutual position of persons taking part in a shooting incident and so the reconstruction of the incident taking into account the chemical examination of the revealed gunshot residues. However, the first publications on the expansion of gunshot residue and the possible range of distance they may reach starting from the gun muzzle presented visual assessment of the gaseous plum photographed by means of a high speed camera [5]. Later publications focused solely on the numbers of metallic particles and neglected the chemical aspect of their dispersion [6, 7].

From the literature review and the research experiences with the application of the SEM-EDX, the author found that this analytical method could be utilized far more comprehensively than until now and so undertook research aiming not only at the identification of GSR but also towards understanding the relationships between their chemical and morphological properties and their source as well as the mechanisms of their formation and dissipation in the environment. The main aims of the research embraced the following: (i) a group identification of the ammunition type from the examinations of GSR with and without the comparative material from the cartridge case, (ii) dispersion of GSR in time and space (in the nearest vicinity of the shooting gun) for establishing the persistence of particles, shooting range, and possibly the mutual positions of the participants of a shooting incident, and also (iii) the obtainment of data on the prevalence of GSR in various professional environments for the assessment and possibly elimination of the risk of contamination with GSR.

## 2. Materials and Methods

The subjects of the study were inorganic components of the gunshot residue obtained with discharge of selected brands of ammunition suitable for popular firearm of calibres 5.6, 6.35, 7.62, 7.65, and 9 mm. Specimens were collected from various substrates, mainly hands, face, hair, and clothing of shooting and nonshooting persons within a screening research.

The study embraced the chemical composition and the morphological features of metallic particles that are dispersed in the space and time after the use of a firearm. The research was based on thorough working out of the methodology of chemical analysis of GSR particles by means of SEM-EDX technique and the interpretation of the obtained results with the use of statistical methods. An initial study concerned the evaluation of the repeatability of the measurement process and its usefulness in comparison with the manual search. The worked out research procedure is multistage including the collection and preparation of specimens, the measurement procedure, and the representation of the collected data in the form being usable for mathematical and statistical evaluation [3, 8].

### 2.1. Specimen Collection and Preparation

The preparation of the material embraced firstly the collection of a representative sample of microtraces as well as their concentration by means of multiple pressing of a SEM stub with adhesive tabs to a surface of interest. Aluminium stubs with adhesive carbon tabs by TAAB Laboratories Equipment Ltd., Berks, UK, were used. About 100 dubbings to the surfaces of interest were made with each stub. The collected specimens were covered with a conductive graphite layer using a SCD 050 sputter, BAL-TECH, Lichtenstein, and a carbon thread (TAAB Laboratories Equipment Ltd, Berks, UK) to prevent the electric charging of nonconductive particles of materials, such as fragments of epidermis, hair, and textile fibres, that usually accompany the metallic particles of interest being collected from various substrates. This way the electric conductivity is being assured to obtain electron images as well the X-ray spectra of good quality. It also prevents accumulating of thermal energy by the adhesive material, which otherwise would soften and immerse the particles, being equivalent to the loss of the evidence material. The last phenomenon was unexpectedly encountered by the author, while analysing for few hours an uncovered specimen with GSR particles that gradually swamped and soon were not detectable. This was similar to the observed softening of the adhesive material of the polymer tab under a light microscope, while preparing other materials for SEM examinations.

### 2.2. Working Out the Procedure of Measurement

The collected specimens were examined by means of a scanning electron microscope JSM-5800 (Jeol Ltd., Tokyo, Japan) coupled with an energy dispersive X-ray spectrometer (Link ISIS 300, Oxford Instruments Ltd., Si/Li detector, ATW—atmospheric thin window, resolution 131 eV for MnK*α* at 10000 counts) or with an energy dispersive X-ray spectrometer and Inca Energy Software rev. 4.09 (Oxford Instruments Ltd., High Wycombe, UK; Si/Li detector, ATW—atmospheric thin window, resolution 133 eV for MnK*α* at 10000 counts). The identification of GSR was performed in an automatic manner with a GunShot or an Inca Feature/GSR programme, Oxford Instruments Ltd.

The measurement process demands performing the following actions: backscattered electron images observation for the location of particles of the expected mean value of the atomic number, collection of X-ray spectrum, and observation of the morphology of each of the individual particles. In contemporary integrated SEM-EDX systems, there is a possibility of automation of a portion of the process. In such a case it embraces the phase of optimisation of the measurement parameters, the automatic search for particles, and finally the manual confirmation of the relevance of the chemical class assigned to a particle with its X-ray spectrum as well the observation of the morphology of single particles.

**Figure 1:**
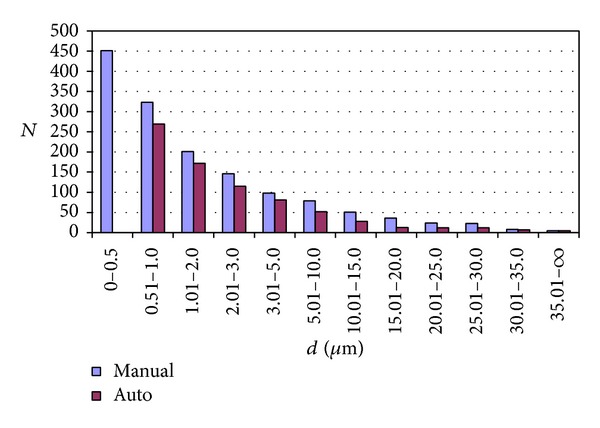
Comparison of the effectiveness of detection of particles in manual and automatic manner. Numbers of particles (*N*) are presented within ranges of effective diameters $d=\sqrt{4S/\pi }$, where *S* is the area of the particle image.

Location of the particles and collection and storage of their X-ray spectra and electron images by means of a manual search are time-consuming and human-engaging and so less economical than the automatic search. The advantage of the automatic method is also the repeatability of the obtained results presented as numbers of particles by means of triple measurement of the same specimen. From the inspection of the results by means of Student's *t*-test, it was found that the measurement is repeatable and precise [8]. Moreover, a comparative analysis of results obtained in both manners: automatic and manual was performed for the same specimen containing a great number of particles (Figure 1). The total number of particles found manually (ca. 1500) was greater than the one found by the programme (ca. 700). However, from the investigation of the distribution of particles against various ranges of their sizes, it turned out that the observed difference resulted from the greater number of particles detected manually in the range of sizes below 1 micrometer (ca. 800), that is, below the limit of detection in the automatic run. Thus, the number of particles within the same range of sizes (ca. 700) was comparable for both methods [8]. This demonstrated an additional advantage of the much faster automatic search for particles.

The worked out research procedure utilising the automatic search for particles guaranteed the detection of particles of the size of at least 1 micrometer due to the appropriate choice of parameters such as the image resolution coupled with the SEM magnification, the accelerating voltage, the beam intensity, and the time of analysis of a single sample stub.

**Figure 2:**
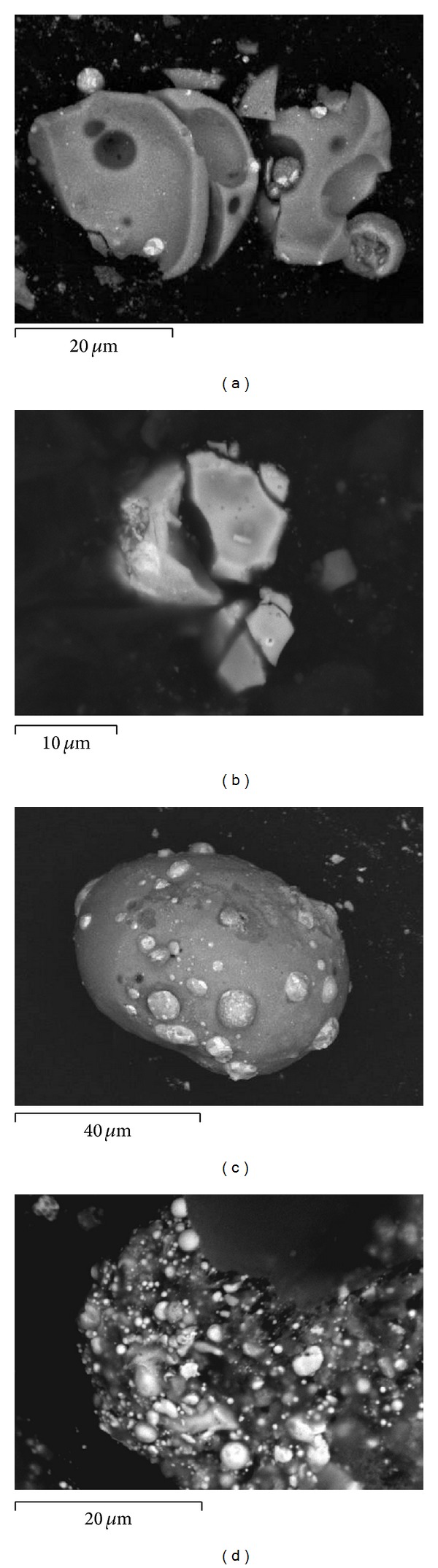
Dynamic effects influencing the numbers of detected particles.

Moreover, Figure 1 shows that a population of GSR particles in the majority consists of particles revealing sizes of about 1 micrometer. Most of them occur isolated in the scanned area of a specimen; however, occasionally, they cluster together forming an aggregate such as the one presented in Figure 2(d).

### 2.3. Methodology of Examinations of the Chemical and Morphological Properties of Populations of Particles

The next important aim of the examinations was working out a method of the analysis of the chemical composition of the whole population of particles detected in a specimen to be able to compare the populations with each other. As a result of a measurement of particular specimens, there are various numbers and various chemical classes as well as various sizes of particles obtained. The variability is a characteristic feature of GSR samples that results mainly from the dynamic conditions of their formation and dissipation as well as from the interactions with the obstacles on their way from the interior of the discharged cartridge case, where they come into being (Figure 2). Much less influence reveals the environmental conditions, individual features of human skin on hands (or of the other substrates), the number of pressings of the stub against the substrate, and also the method of measurement: manual or automatic [3, 8].

To be able to perform comparisons between populations of the detected particles from the chemical point of view, their numbers were transformed into the form of frequencies of occurrence of particles of certain chemical class. It allowed characterizing quantitatively the chemical contents of the whole population of particles in a specimen providing a distribution of detected particles against the chemical contents. This representation of data on the chemical composition of specimens was applied in publications [3, 8–14]. Similar data representation was utilised in research concerning various ranges of the effective diameters of particles as the measure of their morphology in a specimen [12–14] is described later.

Results of the analysis of 135 specimens originating from that same ammunition batch—being obtained during a study on the persistence of particles on various substrates [14]—became an occasion for observation of a number of interesting phenomena that add to the variability of the numbers of the detected particles. One of them was an interaction of particles with the substrate resulting in braking of particle into fragments in the form of polygonal plates. Inspection into each particular plate separately usually leads to its exclusion from the population of GSR taking into account the morphological criterion. Another observed effect was merging of two or more droplets of molten materials or immersion of numerous small metallic particles (of sizes usually below 1 micrometer, i.e., below the limit of detection of the search program) in material consisting of elements of low atomic numbers, such as carbon (*Z* = 6), forming one large particle of about several dozens or several hundreds of micrometers in size. In such cases the program registers a significant number of all the particles; however, it is in the gesture of the analyst to account it as one complex particle or a great number of small particles (Figure 2).

It is worthwhile underlying that the total numbers of the particles detected in various specimens originating from using cartridges of the same ammunition load reveal a significant variability, whereas other parameters describing the population of detected particles demonstrate a good repeatability. This was proved while studying the distributions of particles originating from two types of 9 mm ammunition by Mesko Metal Works, Skarżysko-Kamienna, Poland, performing the analysis of variance of some parameters such as the average value of the effective diameter of particles and a parameter binding the particles diameter and their density is estimated as an arithmetic mean value of the densities of their components [12, 13].

Data in the form of the frequencies of occurrence of particles, in particular chemical classes, were applied in comparisons between specimens originating from the use of, physically, the same ammunition cartridge as well as between samples originating from several cartridges of the same load of ammunition. For this purpose, rang correlation tests: *R*-Spearman and *τ*-Kendall, were applied. Both tests demonstrated agreement in the chemical contents between the analysed specimens and so confirmed the correctness of the worked out and applied analytical procedures within the research of metallic particles [3, 8].

## 3. Results and Discussion

The worked out research methodology provided bases for inspection into the correlations between the chemical composition and parameters describing morphological features of the populations of particles in the dependence on various factors, such as the type of ammunition, the distance from the gun muzzle, the kind of the substrates the particles were deposited on, and the time since shooting.

### 3.1. The Dependence of the Chemical Contents of Particles on the Kind of Ammunition

Systematic studies of metallic particles originating from 6 popular types of ammunition: Luger 9 mm, Makarov 9 mm, (Mesko Metal Works, Skarżysko-Kamienna, Poland); Tokarev 7.62 mm, Margolin 5.6 mm (Tula Cartridge Works, Tula, Russia); Browning 6.35 mm and Browning 7.65 mm (Sellier & Bellot, Vlašim, Czech Republic), were performed [8]. The investigative materials were specimens collected from hands of the shooters immediately after performing the test shots. The collected specimens were examined in the automatic manner by means of the program searching for particles of the predefined features according to the previously described analytical procedure. Thus, the subjects of interest were particles containing one of the seven classes being the combination of the following chemical elements: lead, antimony, and barium and revealing sizes in the range of micrometers. The obtained results were pair-wise subjected to nonparametric test, that is, Spearman and Kendall rank correlation tests. Both of them revealed significant differences among the frequencies of occurrence of particles of particular chemical classes present in the sample of Browning 7.65 mm ammunition from these originating from all the other five examined types of ammunition demonstrating the existence of correlations between certain type of ammunition and metallic particles originating from its discharge.

For data obtained for samples collected during repetitions of test shots with the same type of ammunition (10 samples for Makarov 9 mm ammunition by Mesko Metal Works, Skarżysko-Kamienna, Poland), despite the differences in total numbers of the detected particles, there were observed similarities in proportions of the particular chemical classes of particles that were also confirmed by the application of the Spearman and Kendall rank correlations tests [3].

In the next stage the examinations of samples of particles obtained by repetitions of experimental shots using selected brands of ammunition (Luger 9 mm, Makarov 9 mm, Tokarev 7.62 mm and Browning 7.65 mm) were performed. The obtained results of the chemical analysis of specimens were subjected to a cluster analysis with the Ward's method that revealed clustering of the samples of GSR according to the type of ammunition they originated from [9, 10]. Moreover, taking into account discriminative features established by means of the Mann-Whitney test, the author proposed a classification scheme of particles originating from the four ammunition types into one of three classes. Samples originating from Makarov 9 mm and Tokarev 7.62 mm ammunition revealed a similarity and so created one cluster [10].

The differentiation of populations of metallic particles in the dependence on the type of ammunition, from which they originate, inspired further systematic examinations of gunshot residues originating from the same ammunition type—Luger 9 mm—of various makes and producers. 15 types of the ammunition were selected for the investigations: 8 of traditional and 7 of lead-free ones. Samples of particles were obtained for examinations using two pistols, each placed in especially constructed holder for the gun and four stubs: one placed 10 cm left from the gun near the ejector, one in front of the muzzle in the distance of 10, and two in front of the muzzle in the distance of 40 cm. The experiments were performed in two independent shooting galleries in Wiesbaden and Krakow.

**Figure 3:**
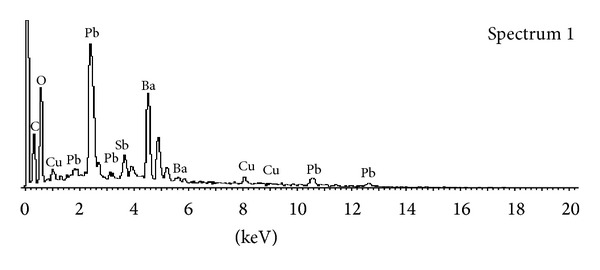
Spectrum of a three-component particle containing traces of copper originating from the cartridge case. The vertical axis represents the intensity of X-ray radiation given in pulse counts (cts); full scale 560 cts.

The sets of specimens collected in the nearest vicinity of the gun were analyzed by means of SEM-EDX method in four laboratories using five different systems of various manufactures. The analytical results obtained for all of the selected ammunition brands of Luger 9 mm ammunition were compared with these obtained in similar manner in the other laboratories using different SEM-EDX systems. The subjects of the statistical analysis were X-ray spectra of at least one thousand particles per ammunition type but not chemical classes as in the previous studies. This attempt was selected to avoid the need of manual check of X-ray spectrum of each particle for confirmation of its chemical content and so making the study more objective. Moreover, X-ray spectra usually include characteristic X-ray lines not only of elements of the primer, but also of elements of the other parts of a cartridge, for example, copper (Figure 3). The idea of taking into account the elements that are present in the cartridge case, the projectile, and the gun that remain in contact with the reacting explosives, in addition to the elements of the primer, resulted from the previous study as one of possible ways of increasing of the discrimination power of the GSR populations originating from various ammunition brands [10]. Since it was necessary to operate on great matrices of data (1000 of X-ray spectra × 15 kinds of ammunition × 2000 points per spectrum), data reduction was performed by means of fast Fourier transform (512 coefficients). The classification of particles into one ammunition type (one of the 15 classes) was achieved by means of the regularized discriminant analysis (RDA). The risk of a misclassification of a single particle to certain ammunition was assessed as about 10% in the model for all 15 ammunition classes and about 2% in the case of the 7 classes of lead-free ammunitions [15]. The obtained results were comparable for all of the applied analytical SEM-EDX systems as well as for both items of firearm that demonstrated an insignificant influence of factors other than the chemical composition of the primer on the elemental contents of the particles, as observed earlier [3, 8–10]. In casework, however, the presence of material remaining inside the gun barrel after previous shots ought to be taken into account, while interpreting the detected particles [11].

Results of the research on differentiation of gunshot particles originating from various types of ammunition demonstrated the direct dependence of their elemental contents on the chemical composition of the ammunition primer [3, 8–10, 15]. The performed examinations were also an occasion to perform a systematic review of the primer mixtures currently used by the ammunition producers. This was of great practical importance as the comprehensive information in this subject collected by Wallace was published later [16]. In the case of ammunition of the chemical content being different from the traditional one, based on lead, antimony, and barium compounds, the commonly accepted classification scheme of GSR may fail in lowering the evidential value of the particles and so, it should be applied with criticism. The study also demonstrated the possibility of a group identification of the residues from their chemical analysis. Although not always a population of particles can be assigned to certain ammunition type and calibre, it is possible to carry out a comparative study to either confirm or exclude whether two samples of GSR originated from the same source.

### 3.2. A Comparative Study of the Chemical Contents and Morphology of Airborne Particles and the Ones Remaining inside the Cartridge Case

Comparative studies of the chemical content and the morphological features of gunshot residue collected from the shooter's hands and from the interior of the cartridge case of physically the same cartridge of Luger 9 mm ammunition were performed. Four types of this ammunition representing three types of primer mixtures were chosen [12]. The subjects of the study were particles originating from the use of ammunition produced by Mesko Metal Works, Skarżysko-Kamienna, Poland (the primer containing mercury fulminate as the initiator, potassium chlorate as the oxidizer and antimony sulphide in the role of the fuel), Sellier & Bellot, Vlašim, Czech Republic (S&B—the primer containing lead styphnate or lead azide as the initiator, barium nitrate—the oxidizer and calcium as the fuel), as well as of the lead-free ammunition of the* sintox* type by Dynamit Nobel, Troisdorf, Germany (DN—containing tetrazene, diazadinitrophenol, powdered titanium and zinc peroxide). The last ammunition was used in two variants: with copper and tin jacketed projectiles.

**Figure 4:**
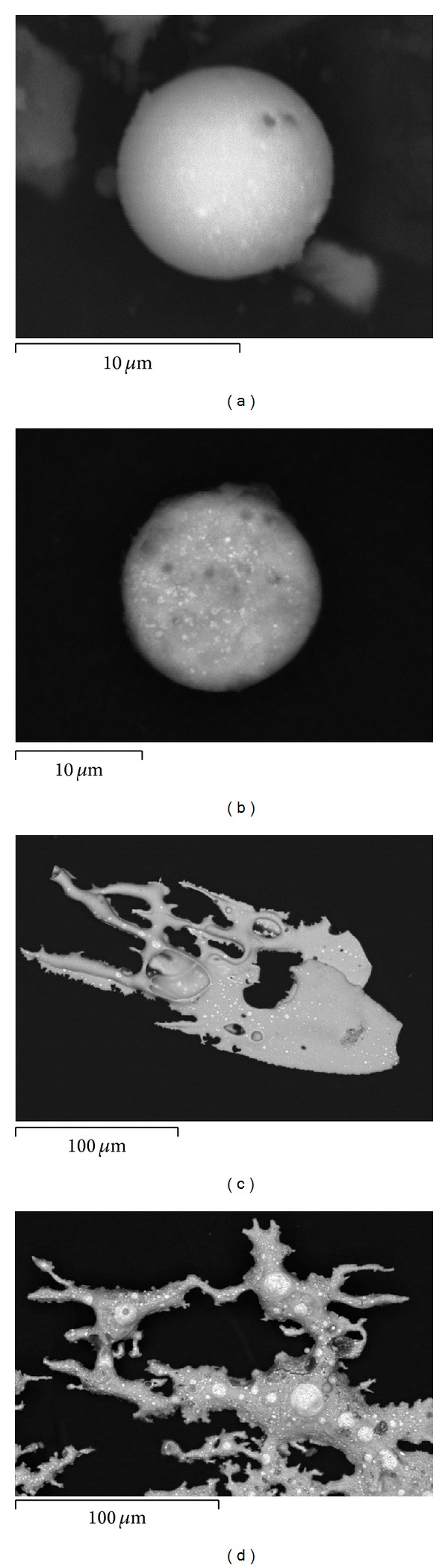
Typical morphology of GSR particles: airborne (a, b) and remaining inside the cartridge case (c, d).

Contrary to common opinions, it was established that there may occur significant differences in the chemical composition, both qualitative and quantitative, between the residue settled on the shooter's hands and these remaining inside the cartridge cases. Whereas in the case of using a S&B ammunition and DN with copper jacketed projectile one can observe the same qualitative chemical contents of particles settled on the shooter's hands and in the projectile, in the case of Mesko and DN with tin jacketed projectile ammunition there are present some similarities as well as significant differences both in the qualitative and quantitative chemical composition of the particles taken from the shooter's hands and the ones remaining inside the cartridge case. The elemental contents of particles present in the interior of the used cartridge case remain in agreement with the elemental contents of the primer mixture. But the elemental contents of particles leaving the gun barrel can be enriched with elements present in the projectile core (e.g., lead in the case of Mesko ammunition) or the projectile jacket (e.g., tin in the case of DN). There are also present differences in the morphological features of the compared residue (Figure 4). When the process of formation of metallic particles completes in the air, they preserve their spherical shape. However, when they do not solidify before the collision, for example, with the interior walls of the cartridge case, they reveal more complex and flat shapes and also usually greater sizes. Neglecting the possible differences of particles taken from the two substrates may lead to a false differentiation of the samples and so a wrong elimination of the suspect taking part in the shooting incident.

The performed examinations allowed also drawing more general conclusions, among which the most important is that the processes of formation and propagation of particles in the cartridge case and inside the barrel proceed in one direction, which causes that the formed GSR particles originating from the primer may join and contribute fragments of the cartridge case, the core and jacket of the projectile, and also the interior walls of the barrel (together with all the impurities present there), and disperse in the surroundings of the shooting gun together with the plum of gases. The possibility of the materials—mainly metals—building the particular components of a cartridge undergoing an interaction with the reacting explosives depends on the physical and chemical properties of the appropriate metals. The correctness of the conclusion was proved in the next stage of the research on the dispersion of metallic particles in the vicinity of the shooting person.

### 3.3. Dispersion of the Metallic Particles in the Surroundings of a Gun

A lack of literature data on the influence of the shooting distance and the kind of the substrate, where the GSR are being collected, on the numbers, chemical composition, and morphological features of the detected particles inspired a systematic research of GSR samples obtained during test shots using two types of full metal jacket (FMJ) ammunition produced by Mesko: Makarov 9 mm and Luger 9 mm ammunition. Both kinds of ammunition were primed with the mixture based on mercury fulminate as the initiator, potassium chlorate as the oxidiser, and antimony sulphide as the fuel [13, 14]. Specimens were collected from targets placed in the following distances from the muzzle: 10, 20, 30, 50, 70, and 100 cm as well as from hands and clothing of the shooter, that is, from places situated in front of and behind the gun muzzle. While performing the test shots, the shooters wore cotton lab coats and the targets were covered either with cotton fabric, or cow's leather simulating popular types of clothing. The experiments were performed in such a manner that three repetitions were obtained for each point chosen in the direction of shooting and the opposite one to evaluate the repeatability of the parameters describing the populations of particles, that is, their numbers, chemical contents, and morphological features represented by the effective diameters that are being obtained by means of SEM-EDX method.

Changes of the numbers, the elemental contents, and the effective diameters of particles with the distance from the muzzle demonstrated that the distribution of particles on the shooter and his nearest vicinity is not uniform, not only from the point of view of the numbers of particles, as expected, but also from the point of view of their chemical composition and dimensions.

Whereas in the direction of shooting a relative decrease of the frequency of occurrence of lead containing particles with the increase of the distance was observed, on the shooting person, that is, in the direction opposite to shooting, a relative increase of the frequency of occurrence of lead containing particles with the increase of the distance from the muzzle took place. The frequency of occurrence of antimony particles revealed an opposite course, that is, a relative increase in the direction of shooting and a relative decrease in the opposite direction. This regularity occurred for both of the examined types of ammunition [13, 14]. As established from the examinations described in the previous chapter [12], lead is detected in the metallic particles originated from the core of the projectile.

The average values of the effective diameters of particles in samples collected from the targets and from the shooter's hands and garment increased with the increase of the distance from the muzzle. Moreover, the values of the effective diameters of particles present in samples taken from the shooter's hands were for both ammunitions significantly greater than for the sample collected from the target in the distance of 10 cm from the muzzle [13, 14].

**Figure 5:**
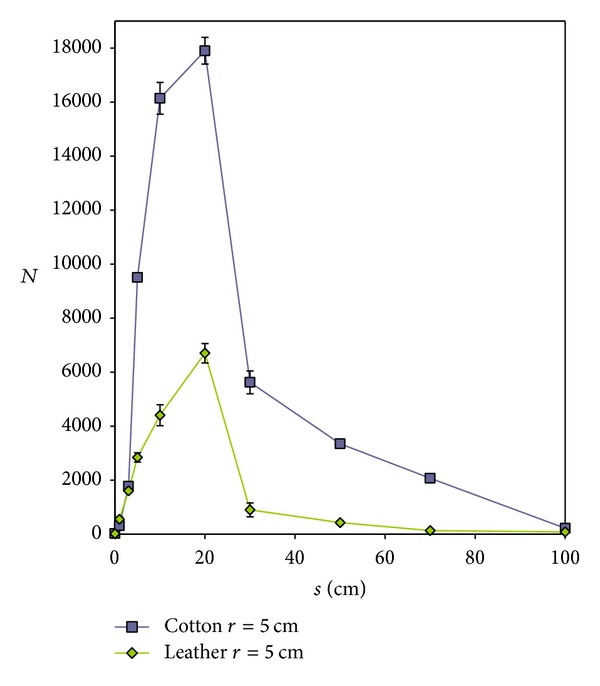
Number of particles (*N*) collected from targets depending on the shooting distance (*s*).

It was also demonstrated that the number of particles settled on certain substrate is dependent not only on the shooting distance and the physical and chemical properties of particles but also on the material of the substrate [14]. Usually more particles are being settled on the cotton fabric than on the cow's leather of the popular leather jackets (Figure 5). This depends on the differences in the adhesion forces acting between the metallic particles and the substrate materials. Moreover, the cotton fabric reveals a more loose structure than the leather, but much more developed surface. Fibres protruding from the threads and the surfaces of the fabric may cause the spherical metallic particle to adhere to the substrate surface in more than one point of contact. Opposite to that, the cow's leather has more compact structure and smoother surface despite of the presence of grooves.

Moreover, it was observed that less lead particles remain settled on the cow's leather than on the cotton fabric [14]. This state may be explained not only with the different properties of the two substrates but also with the great density of lead (11340 kg/m^3^)—nearly twice as big as that of antimony (6697 kg/m^3^). Among lead and antimony particles of the same sizes and similar initial velocity, lead particles reveal a greater mass and so their kinetic energy may exceed the values of energy being appropriate to be able to adhere and stay at the surface of leather substrate.

The relatively great total numbers of metallic particles revealed in samples collected from targets in the distances of about 50–100 cm, when no sooth and unburned propellant grains are being observed around the gunshot hole, enabled establishing an additional category of the shooting distance assessment taking into account physical and chemical examinations of gunshot residue pattern around gunshot hole in the target [14].

Analysing the obtained results, it was found that the process of formation of particles starts with the detonation of the primer and continues at further stages of the gunshot. Together with the gaseous products of the propellant deflagrating, layer by layer metallic droplets move along the walls of the cartridge case. Those of particles that are present in the head of the shock wave reach the bottom of the projectile with the open lead core and may get enriched with this element. Before the projectile is being released from the cartridge case under the growing pressure of gases, a local melting of lead in the surface layer of the core of projectile takes place and the occurring droplets and particles of lead are being pushed aside by the central stream of gases, that is, towards the edges of projectile and subsequently towards the walls of the barrel and the edges of the muzzle. In the moment, when the projectile leaves the gun muzzle, the expanding in each direction gases spread out together with the droplets of rapidly solidifying metals, mostly lead. Thus, relatively great numbers of lead particles are settled on close range targets (10–20 cm) and on the shooter, that is, his garment (in the area of arms and the upper part of the torso) [12–14], as well as his face [17].

From the fact that within the examined metallic particles no spherical particles of copper were encountered, which would have originated from the cartridge case and the projectile jacket, but instead of this lead particles from the projectile core occurred as a result of the interaction of its bottom with the products of the reaction of the primer and the propellant, it was concluded that possible sputtering of the materials of the cartridge case and the projectile core and jacket depends on the physical properties of the constituting metals such as melting point and the specific heat. For an example, the melting point of lead equals 328°C and that of copper equals 1085°C. The specific heat (at 25°C) of lead and copper is 129 and 385 J/(kg K), respectively, which would explain the greatest susceptibility of lead to local melting and getting abstracted from the surface of the uncovered projectile core than that of copper from the cartridge case or the projectile jacket.

The differences between the elemental contents and the morphological features of particles moving into direction of shooting and these of particles moving in the opposite direction give rise to more detailed inferences on the mutual position of the persons taking part in a shooting incident.

### 3.4. Dispersion of the Metallic Particles in Time after Using a Firearm

Comprehensive examinations of the kinetics of the loss of GSR collected simultaneously from hands, face, and clothing of the shooter by means of the assessment of their half-life times as well as establishing the influence of the chemical composition of particles and their morphology on the process was performed [17]. In the examinations, Luger 9 mm ammunition by Czech factory Sellier and Bellot was used, which was primed with lead styphnate or lead azide as the initiator, barium nitrate—the oxidiser—and antimony sulphide in the role of the fuel. This ammunition has guaranteed the presence of the three-components: lead, antimony, and barium particles Pb-Sb-Ba in the investigative material. In the experiment, 5 persons took part and the test shots were performed in 9 attempts so that data were obtained for 30-minute intervals in the range of 0–4 hours. For the obtained data, being the average value of the numbers of particles revealed in 5 specimens (for 5 shooters) there were performed calculations of the half-life times of particles present on the three substrates: (i) hands, (ii) face and hair, and (iii) clothing of the shooting person. The resulting half-life time of Pb-Sb-Ba particles was about 30 minutes for hands, about 60 minutes for clothing, and about 140 minutes for the sample collected from the face and hair of the shooter. The half-life time of all particles was about 50 minutes for hands, about 60 minutes for clothing, and about 170 minutes for the sample collected from the face and hair of the shooter.

**Figure 6:**
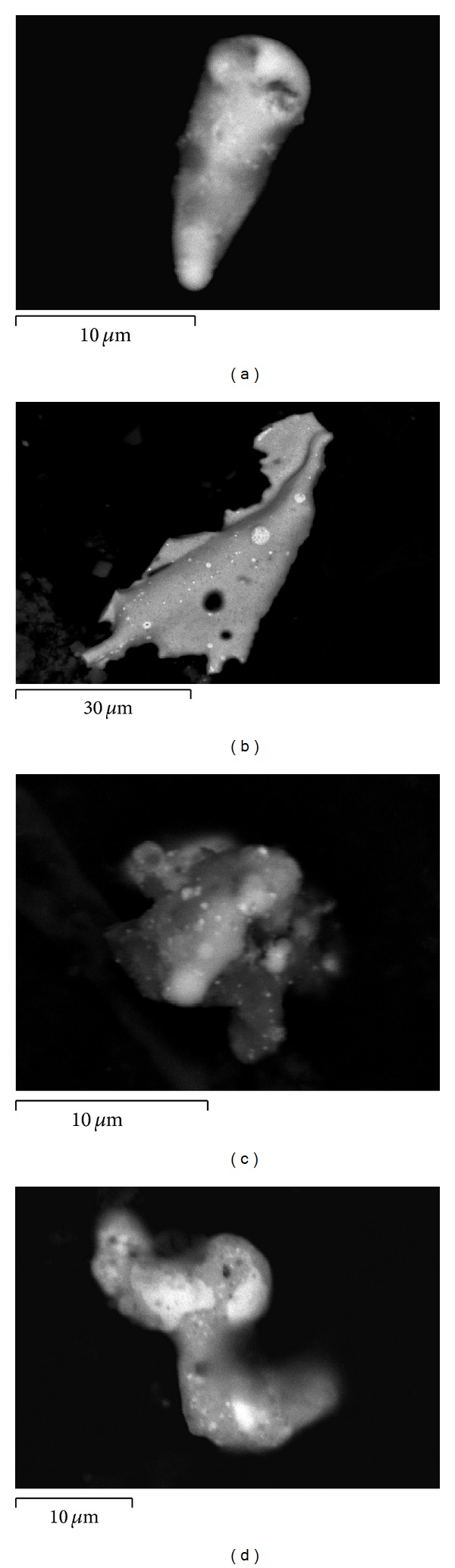
Shapes of the most persistent particles.

Moreover, from the inspection into the frequencies of occurrence of particles of particular chemical classes being the combinations of lead, antimony, and barium in the function of time, it was established that the chemical composition of particles did not reveal significant dependence on time of collecting samples after shooting. However, in samples of particles collected in longer intervals after shooting a relatively greater contribution of particles of the diameters, more than 1 micrometer occurred in the whole population and their shape significantly differed from the spherical one (Figure 6).

From the performed examinations one can conclude that the metallic particles reveal much greater half-life time for specimens collected from the face and hair as well as from the clothing of the shooter than from the person's hands. Thus, it is appropriate to formulate a recommendation that, in addition to samples from hands of the suspects, samples collected from their face and hair as well as clothing should be included into the evidential material, in particular, when more than 4 hours passed between the incident and the collection of the evidence.

### 3.5. The Assessment of the Risk of Misidentification of Gunshot Residue

Results of the research on the kinetics of the loss of GSR particles from the shooting person convince one that the particles are related to the place and time of using a firearm. However, relating an individual with the fact of using a firearm demands not only providing the number, chemical contents, and morphology of the particles detected in the evidence material collected from the person, but also assessing their evidential value. A robust assessment of the evidential value of GSR demands the evaluation of the risk of random contamination with these particles of the selected professional environments due to their primary and secondary transfer. A lack of sufficient information related to prevalence of GSR particles in Poland inspired the screening research performed by the author [18, 19].

In specimens taken from hands of 100 people declaring no contact with firearms, only one spherical particle containing lead, antimony, and barium was found. Numbers of particles found in specimens collected from hands of 50 shooters varied from zero to numbers greater than 100 and were strongly correlated with the time interval between last shooting and collecting specimens. With the application of the criterion of low risk of contamination of hands with GSR, that is, 5 hours after last shooting or handling a gun, very low numbers of GSR particles were encountered among the firearm users. Sixteen out of 17 specimens taken from hands of police officers (94%) and 10 out of 14 of hunters (71%) were free from spherical Pb-Sb-Ba particles. Moreover, the potential secondary transfer was traced on the example of families of 5 hunters as well as chosen activities, such as examinations of short-range gunshot damages in clothing, reloading a gun, or collection of specimens from shooters. All these have shown that the level of secondary transfer might be significant and depends mainly on the recent taking part in shooting activity and its frequency. The obtained results remain in agreement with some mocked situations of the secondary transfer studied by French at al. [20].

Results of the study allowed formulating a recommendation that crime scene officer, who is to collect evidence microtraces from suspects, should not be a user of firearms, or should collect a control sample from his/her hands prior to the evidence collection—a procedure for prevention from contamination of the evidence material at its collection.

Also a potential risk of contamination with the characteristic GSR particles of the laboratory housing the SEM-EDX analytical system serving to the GSR examinations for forensic expertise was evaluated. Analysing 55 specimens of microtraces collected in various time intervals, no particles of the chemical contents and morphology of gunshot residue were revealed. That confirmed the procedures of secure handling of the evidence material for GSR search to be appropriate and preventing from the potential contamination [18, 19].

From the performed research one can conclude that the greater the number of revealed particles the greater their evidential value in the aspect of the relation of an individual with a shooting incident. The recommendation on the securing clothing of the suspects due to a rapid loss of the GSR particles from people's hands appears to be right also for increasing the possibility of detection of a greater number of particles, when examining more evidence items. The value of analytical findings as a group of particles revealing defined chemical and morphological characteristics rather than solely their number was also demonstrated by Gauriot et al. [21].

Evaluation of shooting incidents with the use of modern less toxic ammunition types that produce less characteristic primer residue may demand an application of techniques complementary to SEM-EDX, such as integrated ion beam analysis (IBA) for examinations of inorganic component [22, 23], or variety of spectrometric and chromatographic methods for analysis of organic gunshot residues [24–27].

## 4. Summary

This paper presents a review of chosen aspects of examinations of inorganic gunshot particles by means of SEM-EDX technique. In the first stage, original research methodology of gunshot particles was worked out, which embraced not only a precise and repeatable procedure of detection and identification of particles in the automatic manner, but also a representation of the obtained analytical data in the form of the frequencies of occurrence of particles of certain chemical class (or a class of certain range of the effective diameters) within the whole population of particles revealed in a specimen.

On the basis of this methodology, some relationships were established between the chemical and morphological properties of populations of particles and factors, such as the type of ammunition, the distance from the gun muzzle to the target, the type of a substrate on which the particle sediments, and the time between shooting and collecting the specimens.

Significant differences in the chemical composition of GSR originating from various types of ammunition, depending mainly on the composition of the primer mixture, were established. This gave rise to working out the method of group identification of ammunition used in a shooting incident, when the cartridge case is not available for examinations.

Chemical and morphological examinations of particles collected from the shooter's hands from the interior of, physically, the same cartridge revealed either similarities or discrepancies depending on the chemical composition not only of the primer, but also other parts of the cartridge. This ought to be taken into account in the expert's report for not falsely excluding the possibility of a suspect to be a partaker in a shooting incident.

It has been established that the distribution of particles in the surroundings of the shooting gun is not uniform. The numbers of particles, their chemical composition, and their morphological features depend on the distance from the muzzle of the shooting gun and the type of the substrate the particles sediment on. Results of this study gave rise to working out the method of shooting distance estimation from the physical and chemical examinations of GSR pattern around the gunshot wounds and damages—extending the possibilities of shooting distance estimation with range of about 50–100 cm, in addition to the three categories commonly used until now: (i) contact or a nearest vicinity shot (about 0-1 cm), (ii) close distance shot (about 1–50 cm), and (iii) distant shot.

A consistency of the elemental composition of the residue remaining inside the cartridge case with that of the primer was observed, whereas the elemental contents of the airborne particles (e.g., collected from the shooter's hands) differed. That was most probably resulting from the interactions of the products of the reaction of the primer and the propellant with other parts of the ammunition (i.e., materials of cartridge case, the projectile core, and jacket) as well as the interior surfaces of the gun barrel. Thus, it was established that GSR occurs in the subsequent stages of the explosives reaction of the primer and the propellant propagate inside the cartridge case and the gun barrel only in one direction—towards the muzzle, and so they do not return or mix with each other.

The fact of variability of the chemical composition of particles in the dependence on the distance from the shooting gun, both in the direction of shooting and in the opposite one, could be utilised for establishing the positions of the partakers in the shooting incident in relation to the firearm and so to the direction of shooting.

The kinetics of the loss of GSR particles simultaneously from the shooter's (i) hands, (ii) face and hair as well as (iii) garments was established in the form of the half-life times. It was also found that the chemical composition and sizes of particles reveal practically no influence on the loss of particles, whereas irregularly shaped particles turned out to be more persistent than the regularly spherical ones.

The fundamental information necessary for the assessment of the risk of contamination with GSR particles of selected populations in Poland was obtained and utilised for working out the rules of its elimination or diminishing, while handling the evidence in shooting cases.

Each of the worked out aspects of chemical analysis of metallic particles may significantly contribute to reconstruction of the course of a shooting incident. In spite of the fact that usually the physical and chemical examinations lead to a group identification and are complementary to other types of information included in the cases files, in specific cases they may be decisive in establishing the circumstances of the incident.
